# Visualizing threat and trustworthiness prior beliefs in face perception in high versus low paranoia

**DOI:** 10.1038/s41537-024-00459-z

**Published:** 2024-03-20

**Authors:** Antonia Bott, Hanna C. Steer, Julian L. Faße, Tania M. Lincoln

**Affiliations:** Clinical Psychology and Psychotherapy, Faculty of Psychology and Human Movement Science, Universität Hamburg, Hamburg, Germany

**Keywords:** Human behaviour, Psychosis

## Abstract

Predictive processing accounts of psychosis conceptualize delusions as overly strong learned expectations (prior beliefs) that shape cognition and perception. Paranoia, the most prevalent form of delusions, involves threat prior beliefs that are inherently social. Here, we investigated whether paranoia is related to overly strong threat prior beliefs in face perception. Participants with subclinical levels of high (*n* = 109) versus low (*n* = 111) paranoia viewed face stimuli paired with written descriptions of threatening versus trustworthy behaviors, thereby activating their threat versus trustworthiness prior beliefs. Subsequently, they completed an established social-psychological reverse correlation image classification (RCIC) paradigm. This paradigm used participants’ responses to randomly varying face stimuli to generate individual classification images (ICIs) that intend to visualize either facial prior belief (threat vs. trust). An independent sample (*n* = 76) rated these ICIs as more threatening in the threat compared to the trust condition, validating the causal effect of prior beliefs on face perception. Contrary to expectations derived from predictive processing accounts, there was no evidence for a main effect of paranoia. This finding suggests that paranoia was not related to stronger threat prior beliefs that directly affected face perception, challenging the assumption that paranoid beliefs operate on a perceptual level.

## Introduction

Delusions, characterized as fixed beliefs that persist despite lacking or conflicting evidence^1^, represent a core symptom of schizophrenia and other psychotic disorders. They predominantly revolve around social themes^2^, with the most prevalent type, paranoid delusions, involving the belief that others intend to persecute or harm oneself^3,4^. While manifest paranoid delusions are typically associated with significant distress, they exist on a continuum with milder forms of paranoid beliefs found in the general population^5–7^. Over the past decades, a multitude of theoretical models have proposed various risk factors contributing to the formation of paranoid beliefs, including cognitive reasoning biases^8–10^ and social risk factors^8,9^. Specifically, paranoid beliefs have been consistently associated with premorbid social adversity experiences (e.g., interpersonal childhood trauma)^11,12^, mediated by learned negative beliefs about others^13^. In recent years, our mechanistic understanding of delusions has significantly progressed through the lens of predictive processing accounts. According to this framework, the brain generates predictions about upcoming sensory inputs (*prior beliefs*) and integrates them with the observed sensory inputs to refine future predictions^14–17^. This integration is weighted by the relative certainty (*precision*^18^) assigned to both components. The greater the relative precision assigned to the prior belief, the less the observed inputs impact the final percept (*posterior belief*), and vice versa^16^. A compelling illustration is face pareidolia, where the perception of faces in inanimate objects could be evoked by highly precise prior beliefs for facial features^19,20^. Within the predictive processing framework, delusions are proposed to arise as overly precise prior beliefs formed to resolve the chronic uncertainty associated with sensory inputs^21–24^. Similarly, paranoid delusions can be reconceptualized as precise *threat prior beliefs*, sculpting the individual’s perception as if viewing the world – including other people – through “threat-colored glasses”.

Supporting this conceptualization, individuals with paranoid beliefs have been found to misclassify faces with neutral emotional expressions as angry^25–27^ and rate them as less trustworthy^28–30^ compared to those without such beliefs (but see^31–33^). However, these findings are based on explicit ratings derived from individuals’ *percepts*, thus failing to disentangle the relative impact of prior beliefs and sensory inputs on perception. Previous studies attempting to isolate the impact of prior beliefs on perception in individuals with psychotic symptoms and delusion-proneness utilized various signal detection paradigms^34–39^. These paradigms typically involved the detection of a specific signal within ambiguous non-social stimuli, leveraging experimentally induced prior beliefs to resolve sensory ambiguity. For instance, participants could rely on cues previously associated with leftward versus rightward rotation to detect the ambiguous rotation direction of dot spheres^36^. The severity of psychotic symptoms positively correlated with the reliance on these prior beliefs in determining rotation directions^36^, consistent with the concept of overly precise prior beliefs. However, despite the predominantly social nature of delusional beliefs, investigations into the imbalanced integration during perceptual inference in the social domain are scarce^40–42^. Moreover, existing studies focused on detecting the mere presence of hidden social stimuli, such as a person in a two-tone image^41^ or faces within visual noise patterns^42^. Thus, it remains unclear whether paranoid beliefs condense in overly precise threat prior beliefs, shaping the perception of ambiguous social sensory inputs.

We addressed this objective within the domain of face perception, utilizing the established social-psychological reverse correlation image classification paradigm (RCIC^43,44^; for reviews, see^45,46^). This data-driven signal detection technique enables the visualization of individuals’ mental representations of a face with specific emotional states or traits (e.g., anger, trustworthiness). In the standard RCIC paradigm, participants view pairs of ambiguous faces in a series of trials, selecting the face they deem to best represent a designated state or trait (e.g., “Who is more trustworthy?”). In contrast to traditional signal detection paradigms, all sensory inputs vary randomly (i.e., one constant ‘base face’ is superimposed with two unique random visual noise patterns in each trial). This randomness ensures that stimulus selection is guided by participants’ individual mental representations, such that averaging all selected stimuli produces individual classification images (ICIs) that distill the features guiding their selections^46^. In other words, RCIC offers a nuanced visualization of facial prior beliefs (e.g., of a trustworthy face).

In the present study, we applied a variant of this paradigm^47^ to investigate whether the impact of threat prior beliefs on the perception of ambiguous faces is stronger in individuals with high versus low paranoia. Furthermore, we examined whether this effect is specific to threat prior beliefs or extends to trustworthiness prior beliefs (i.e., a primary dimension in face perception and evaluation with contrasting valence^46,48^). In this variant, participants initially viewed face stimuli labeled as ‘members of two fictitious groups’ (*Group X* and *Group Y*), paired with written descriptions of threatening versus trustworthy behaviors (e.g., “This Group X member spies on you” vs. “This Group Y member keeps a secret you told them”). In a subsequent RCIC paradigm, they selected those faces they deemed most likely to depict *a member of one of these groups* (similar to^47^). As all participants viewed identical face stimuli, any systematic variation in the appearance of the resulting ICIs can be attributed to individual differences in the extent to which facial prior beliefs, activated by the provided behavioral descriptions, affected the perception of ambiguous faces. Consistent with previous findings^47^, we expected the ICIs to appear more threatening following the threat prior activation and more trustworthy following the trustworthiness prior activation (H1). Building on the conceptualization of paranoid beliefs as overly precise threat prior beliefs, we expected individuals with high paranoia to generate ICIs that appear more threatening and less trustworthy as compared to individuals with low paranoia (H2). Additionally, we explored whether social adversity experiences and generalized negative beliefs about others moderated the impact of prior beliefs on face perception. We expected a larger impact of threat prior beliefs on face perception in individuals with high versus low levels of social adversity experiences (H3) and negative beliefs about others (H4).

## Methods

This preregistered study (https://osf.io/epbw3) was approved by the Local Ethics Committee of Universität Hamburg and was conducted in accordance with the Declaration of Helsinki.

### Participants and procedure

**Fig. 1:**
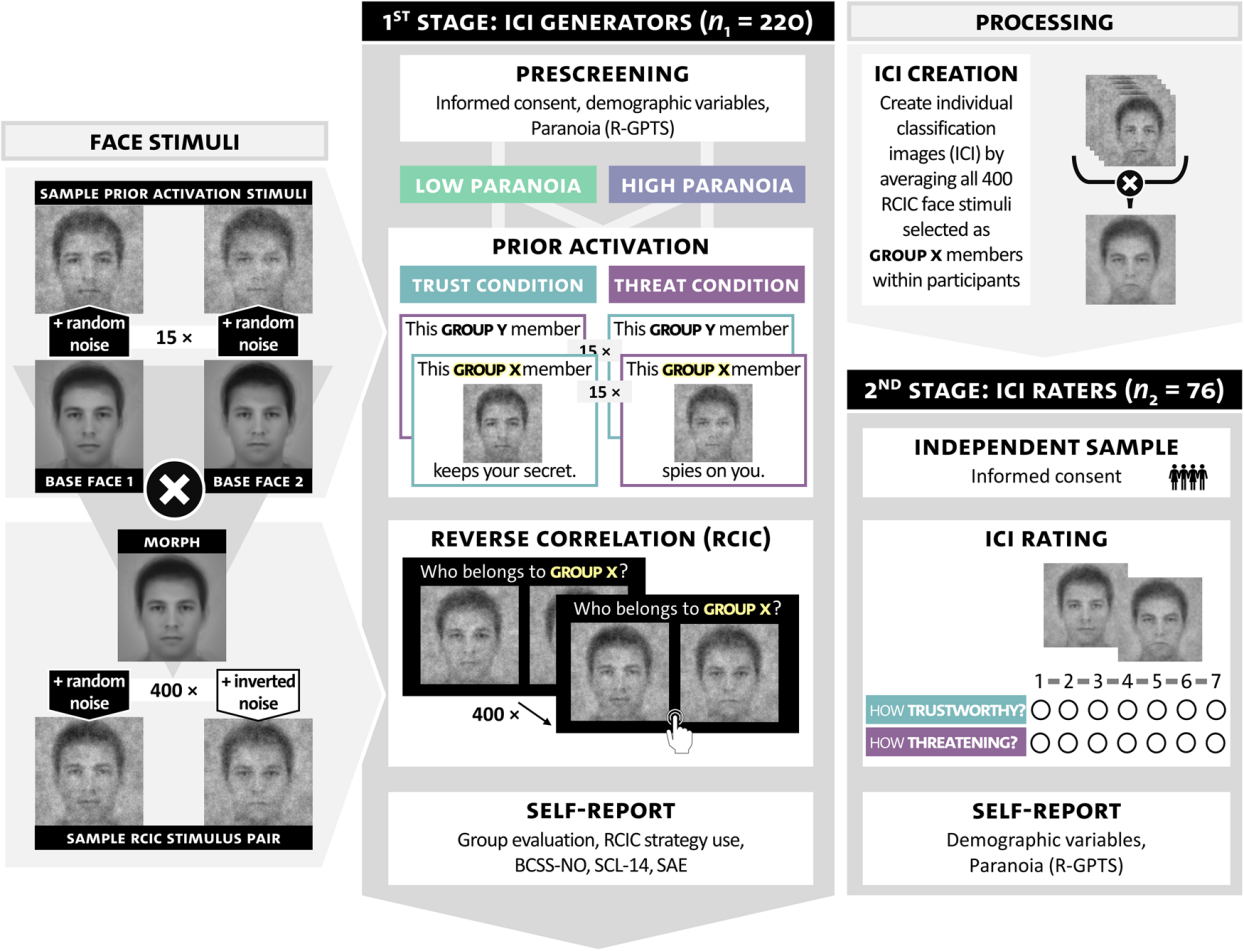
Procedure flow chart and face stimuli used during prior activation and reverse correlation image classification (RCIC). *BCSS-NO* Negative beliefs about others, *ICI* individual classification image, *R-GPTS* Revised Green Paranoid Thoughts Scale, *SAE* Social adversity experiences, *SCL-GSI* Symptom Checklist.

We conducted this study as multi-stage online experiment (see Fig. 1 for an overview). Upon interest, individuals filled out a short online prescreening including demographic variables and self-reported paranoia. Eligible individuals were forwarded to the first stage, comprising a quasi-experimental factorial design with *Paranoia level* (low vs. high) and *Prior activation condition* (trust vs. threat) as between-subjects factors. Following an affective state assessment and a prior activation phase, participants engaged in a RCIC paradigm. Afterwards, they completed their participation by filling out self-report measures. In an interspersed processing stage, we created ICIs from the stimuli selected in the RCIC paradigm. In the second stage of the study, an external sample rated the ICIs on threat and trustworthiness, constituting the primary outcome. Finally, participants from this external sample provided self-reports of paranoia and demographic variables.

Participant recruitment included social media posts, flyers, and advertisements via *Prolific* (www.prolific.com). General inclusion criteria were informed consent, minimum age of 18 years, sufficient self-reported German language skills, normal or corrected eyesight, and participation using a desktop PC or laptop. A lifetime diagnosis of neurological disorders (e.g., prosopagnosia), participation via mobile devices or failing attention checks twice led to exclusion from participation. For sample assignment in the first stage, we used the persecution subscale of the revised Green et al. Paranoid Thoughts Scales (R-GPTS^49,50^). Individuals with a score of ≥11 (i.e., *moderately severe*) and ≤5 (i.e., *within average range*) were assigned to the high paranoia (HP) and low paranoia (LP) sample, respectively. Individuals scoring between 6 and 10 were excluded. An a priori power analysis (G*Power, Version 3.1.9.4^51^) based on α = 0.05 and 1-β = 0.80 in a fixed-effects analysis of covariance (ANCOVA) indicated a required sample size of 220 participants to detect a small to moderate between-subjects effect (Cohen’s *f* = 0.19). Of 279 eligible individuals who started the study, 44 failed to complete it and 11 failed the attention checks. We excluded four additional participants from all analyses (see below). The final sample thus consisted of 220 participants: 109 HP and 111 LP participants. Participants were compensated with EUR 5.80 (£5.00) for participation via Prolific (47.3%) or invited to take part in a lottery (10 online vouchers à EUR 5.00) if they did not wish to participate via Prolific (52.7%). For the second stage of the study, we recruited an external sample of 76 university students in exchange for partial course credit. In addition to the general eligibility criteria above, participants of the first stage were excluded from participation to ensure naivety regarding ICI generation including the design of the first stage.

### Materials

#### Prior activation

We used a counterbalanced block-wise prior activation phase to activate participants’ mental representations (i.e., prior beliefs) of threatening versus trustworthy faces. To this end, we exposed participants to what we called members of two fictitious groups (labeled *Group X* and *Group Y*) by presenting them with differentiable sets of face stimuli paired with unique written behavioral descriptions (15 stimulus pairs per group). For half of the participants, the behavioral descriptions paired with Group X implied the trait *threatening* (e.g., “This Group X member stares at you”), whereas the behavioral descriptions paired with Group Y implied the trait *trustworthy* (e.g., “This Group Y member respects your privacy”). For the other half, this group-trait association was reversed (X = trustworthy, Y = threatening). The face stimuli consisted of one ‘base face’ per group (i.e., morphs of 10 human face images each, randomly selected from the Chicago Face Database^69^) which we converted to grayscale and smoothed with a Gaussian blur. Finally, we superimposed each group-specific base face with 15 unique patterns of visual noise to generate subtle variations of the same underlying face (see Fig. 1). The images did not differ with respect to relevant traits (e.g., threat, trustworthiness, masculinity, attractiveness; see Supplementary Information S1). The assignment of base faces to group, block order (Group X vs. Group Y first), and stimulus order within blocks were randomized. Participants were instructed to carefully read and memorize the presented materials. Task completion was self-paced (*Mdn* = 8.53 min, *SD* = 8.08), with a minimum presentation duration of 5 s per stimulus pair.

#### Reverse correlation image classification (RCIC) paradigm

Following prior activation, participants completed a two-image forced choice RCIC paradigm^43,44,47^ with 400 trials. For this paradigm, we created a perfect morph of the two group-specific base faces to create one group-ambiguous base face (i.e., reflecting both base faces to the same extent; see Fig. 1). Next, we superimposed this morphed base face with both 400 unique random visual noise patterns and their mathematical inverses (i.e., a white pixel in the original noise pattern was black in the inverted pattern and vice versa, see Supplementary Information S1) by using the rcicr^52^ package for R^53^. Thus, each of the 400 stimulus pairs reflected *random and very subtle* variations of the same underlying base face, with anti-correlated variation within stimulus pairs. In each trial, participants were presented with one of these stimulus pairs presented side-by-side against a black background (512 × 512 pixels) and were instructed to select the face they spontaneously deemed most likely to *depict a Group X* member (with “Who belongs to Group X” presented above the stimuli). Note that Group X was either paired with threat-implying behavioral descriptions (*threat* condition) or trustworthiness-implying behavioral descriptions (*trust* condition) during prior activation. Task completion was self-paced (*Mdn* = 16.59 min, *SD* = 13.16). After blocks of 100 trials, participants were offered a short break (30 s).

#### Group evaluation and RCIC strategy use

Following the RCIC paradigm, we measured explicit evaluations of both Group X and Group Y on separate 7-point rating scales (ranging from *−3: very negative* via *0: neutral* to *+3: very positive*). This manipulation check was intended to assess whether the behavioral descriptions induced differently valenced general perceptions of the two fictitious groups. This would be particularly important in the absence of a main effect of condition on ICI appearance (e.g., no threatening appearance in the threat condition), ensuring the strength of the manipulation. Furthermore, we asked participants to report their RCIC selection strategies (see Supplementary Information S2).

### Self-report measures

#### Paranoia

We measured paranoia using the 10-item self-report persecution subscale of the R-GPTS^49^. The R-GPTS assesses to what extent participants have experienced paranoid thoughts during the last month (e.g., “People wanted me to feel threatened, so they stared at me”) on 5-point scales (ranging from *0: not at all* to *4: totally*). The R-GPTS has shown good psychometric properties^49,54,55^ and achieved satisfactory internal consistencies within the present samples (Cronbach’s α = 0.93 and 0.89 for the first and second stage, respectively).

#### Negative affective states

We asked participants to report current feelings of happiness, sadness, anger, shame, and guilt on 5-point scales (ranging from *0: not at all* to *4: very*; based on^56^) before prior activation to control for negative affective states.

#### Negative beliefs about others

We measured beliefs about the self and others with the Brief Core Schema Scales (BCSS^57^). The BCSS is a 24-item self-report instrument with four 6-item subscales assessing positive and negative beliefs about the self (e.g., “I am talented” vs. “I am unloved”) and others (e.g., “Others are fair” vs. “Others are hostile”) on 5-point scales (ranging from *0: no, don’t believe it* to *4: yes, believe it totally*). Only the Negative Others subscale (BCSS-NO) was included in the present analyses. The BCSS demonstrated good psychometric properties^57^.

#### Social adversity

We measured participants’ social adversity experiences with four items assessing the prevalence of emotional, psychological, physical, and sexual abuse prior to their 18^th^ birthday rated on 6-point scales (ranging from *0: never* to *5: very often*; based on^58^).

#### General psychopathology

We administered a 14-item short version of the Symptom-Checklist (SCL-GSI^59^) to control for participants’ general psychopathology (i.e., the severity of phobic, depressive, and somatic symptoms) during the last seven days on 5-point scales (ranging from *0: not at all* to *4: very strong*).

#### ICI creation and ICI rating

We created one ICI per participant by averaging all noise patterns selected during RCIC and by re-superimposing this average onto the morphed base face, using the rcicr package^52^ for R (version 4.1.0^53^). These ICIs can be interpreted as visual proxies of participants’ mental representations of a typical Group X member and reflect the extent to which the behavioral descriptions informed their facial prior beliefs. In the second stage of the study, an external sample rated the ICIs on threat and trustworthiness in random order using separate 7-point scales (ranging from *1: not at all* to *7: very*). Due to the large number of stimuli, ICIs were presented in three approximately equal-sized blocks (i.e., two blocks with 73 and one block with 74 ICIs) and raters could decide how many blocks they wished to rate. ICI order and trait rating order were randomized. Each ICI was rated by 26 participants who were blind to all procedures related to the generation of the ICIs.

### Statistical analysis

We combined participants’ separate explicit group evaluations into a difference score (i.e., positive values = Group X was rated more positively than Group Y; negative values = Group X was rated more negatively than Group Y) and applied non-parametric Wilcoxon rank-sum tests to account for the non-normality of this metric. Due to the high inter-rater reliability of the ICI ratings (*ICC*(1,*k*) = 0.97 for threat and trustworthiness ratings), we averaged them across raters to obtain one mean threat and one mean trustworthiness value per ICI. Because these values were highly correlated (*r* > 0.80), we subtracted the mean trustworthiness from the mean threat ratings (i.e., ICI threat score; positive score = ICI was rated as more threatening than trustworthy; negative score = ICI was rated as more trustworthy than threatening). We excluded four participants from the analyses because they did not comply with the RCIC instructions (*n* = 3) or their ICI was not rated due to a technical error (*n* = 1; see Supplementary Information S3). ICI threat scores were submitted to a 2 (Paranoia: low vs. high) × 2 (Condition: trust vs. threat) between-subjects analysis of variance (ANOVA) to test for main and interaction effects (H1 & H2). In a second step, we added BCSS-NO and social adversity experiences as well as their first- and second-order interactions with the between-subjects factors as covariates (ANCOVA) to explore whether ICI appearance covaried with these variables (H3 & H4). We complemented the frequentist analyses with Bayesian analysis counterparts performed with JASP^60^ according to guidelines^61,62^, and report Bayes Factors (BF) along with the *p*-values. BF hypothesis testing directly and continuously compares two competing statistical models, with BF_10_ quantifying the amount of evidence for the alternative over the null hypothesis and BF_incl_ quantifying the amount of evidence for including a predictor in a model (e.g., ANOVA) over excluding it. A widely accepted rule of thumb^63^ distinguishes ‘anecdotal’ (1 < BF < 3) from ‘moderate’ (3 < BF < 10) and ‘strong’ evidence (BF > 10; see Supplementary Information S4 for a more details).

## Results

### Descriptive statistics and group classification images

**Fig. 2:**
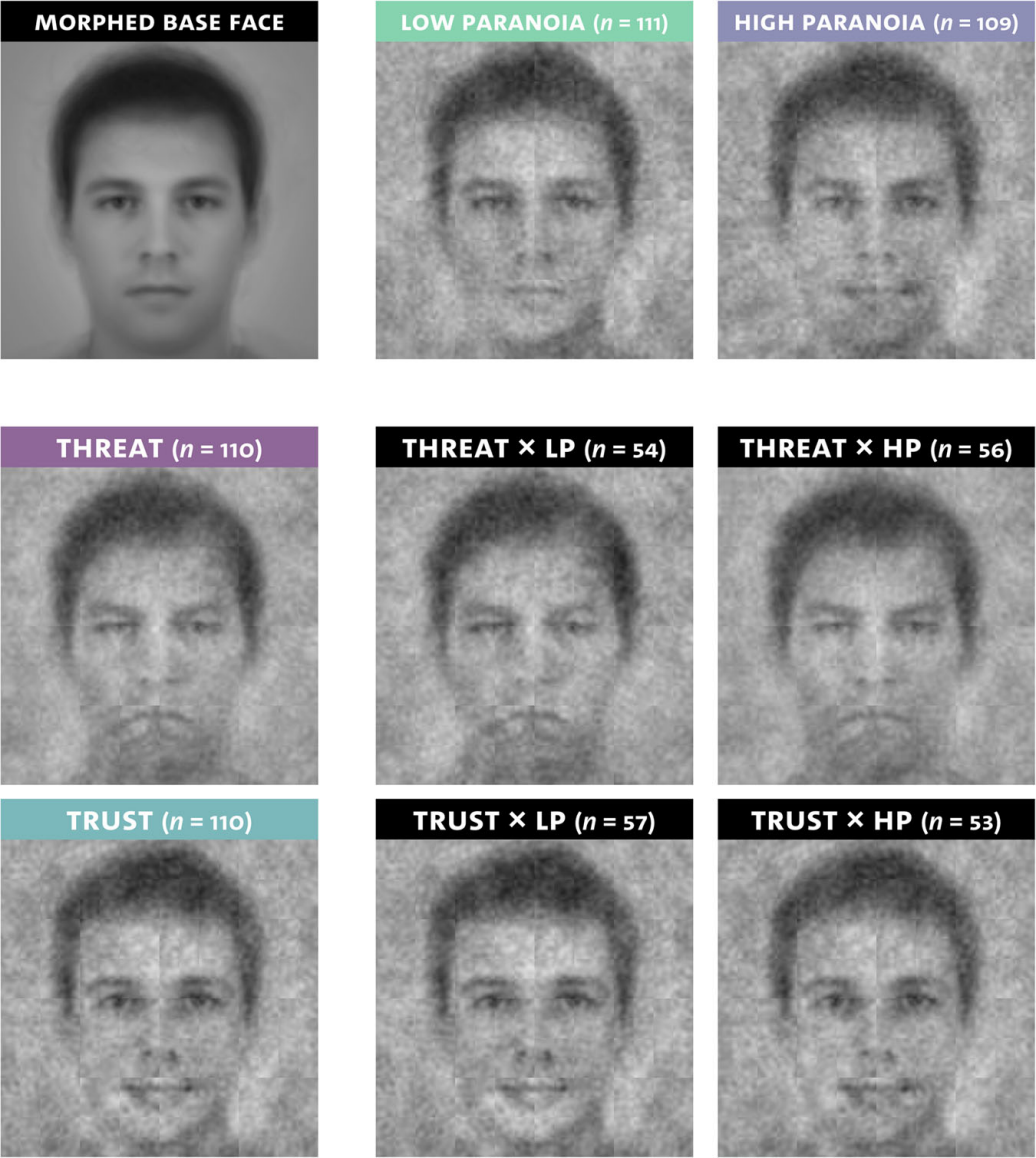
Averaged classification images per condition, paranoia level, and condition×paranoia combinations. Due to an increased type I error rate associated with averaged CI ratings^68^, we restricted our inferential analyses to the ICI ratings. The morphed base face is presented for reference. *HP* high paranoia, *LP* low paranoia.

Socio-demographic characteristics are presented in Table 1. Participants with high versus low paranoia differed significantly with respect to mean age and educational level. Descriptive statistics of ICI threat scores and self-report measures are shown in Table 2. Participants with high versus low paranoia differed significantly with respect to all self-report measures (see Supplementary Information S5). For a visualization of averaged classification images, see Fig. 2.

**Table 1:** Sample characteristics.

	First stage			Second stage
	Low paranoia (*n* = 111)	High paranoia (*n* = 109)	Test statistic	Raters (*n* = 76)
Age, *M* (*SD*)	32.76 (14.26)	26.4 (7.64)	*t*(168.94) = 4.13, *p* < 0.001^b^	25.45 (6.61)
Gender (female/male/diverse)	65/46/0	52/53/4	χ²(1) = 1.43, *p* = 0.232^c^	55/21/0
Education (low/medium/high)	1/9/100^a^	5/21/83	χ²(2) = 9.04, *p* = 0.011	0/2/74
R-GPTS, *M* (*SD*)	0.88 (1.28)	17.21 (5.63)	-	4.88 (6.29)
Number of blocks rated (one/two/three)	-	-	-	58/5/13

*R-GPTS* Revised Green et al. Paranoid Thoughts scale, persecution scale.

^a^*n* = 1 missing due to technical error.

^b^Welch two sample *t*-test.

^c^Diverse gender was omitted from this test due to a limited number of cases.

**Table 2:** Means and standard deviations per paranoia level and condition.

		Low paranoia				High paranoia			
		Trust (*n* = 57)		Threat (*n* = 54)		Trust (*n* = 53)		Threat (*n* = 56)	
Variable	α	*M*	(*SD*)	*M*	(*SD*)	*M*	(*SD*)	*M*	(*SD*)
ICI threat score^a^	-	−0.68	(1.41)	1.50	(1.52)	−0.92	(1.32)	1.07	(1.30)
Explicit group evaluation^b^	-	3.33	(2.32)	−3.59	(2.12)	3.89	(2.34)	−3.95	(1.94)
BCSS-NO	0.87	4.81	(3.75)	4.65	(3.15)	10.00	(4.66)	9.25	(4.28)
SAE	0.76	2.93	(3.35)	2.94	(3.19)	5.40	(4.52)	5.68	(4.06)
State negative affect	0.80	0.33	(0.37)	0.37	(0.43)	0.98	(0.85)	0.71	(0.70)
SCL-GSI	0.91	7.75	(7.50)	7.31	(5.99)	17.91	(10.79)	14.27	(10.70)

*ICI* Individual classification image, *BCSS-NO* Brief Core Schema Scale-Negative Others, *SAE* Social Adversity Experiences, *SCL-GSI* Symptom Checklist (general severity index), *α* Cronbach’s α.

^a^Difference score (mean threat rating – mean trustworthiness rating).

^b^Difference score (explicit Group X evaluation – explicit Group Y evaluation).

### Manipulation check

As expected, there was strong evidence that participants in the threat condition explicitly evaluated Group X significantly more negatively than Group Y (*M* = −3.77, *SD* = 2.03), while the opposite was true for participants in the trust condition (*M* = 3.60, *SD* = 2.34; *W* = 411.00, *p* < 0.001, **BF**_**10**_ = 3.48$\times$10^8^). Moreover, explicit evaluations did not differ across paranoia levels (HP: *M* = −0.14, *SD* = 4.48; LP: *M* = −0.04, *SD* = 4.13; *W* = 5951.00, *p* = 0.834, **BF**_**10**_ = 0.15), indicating that the prior activation was equally effective across samples.

### ICI ratings

**Fig. 3:**
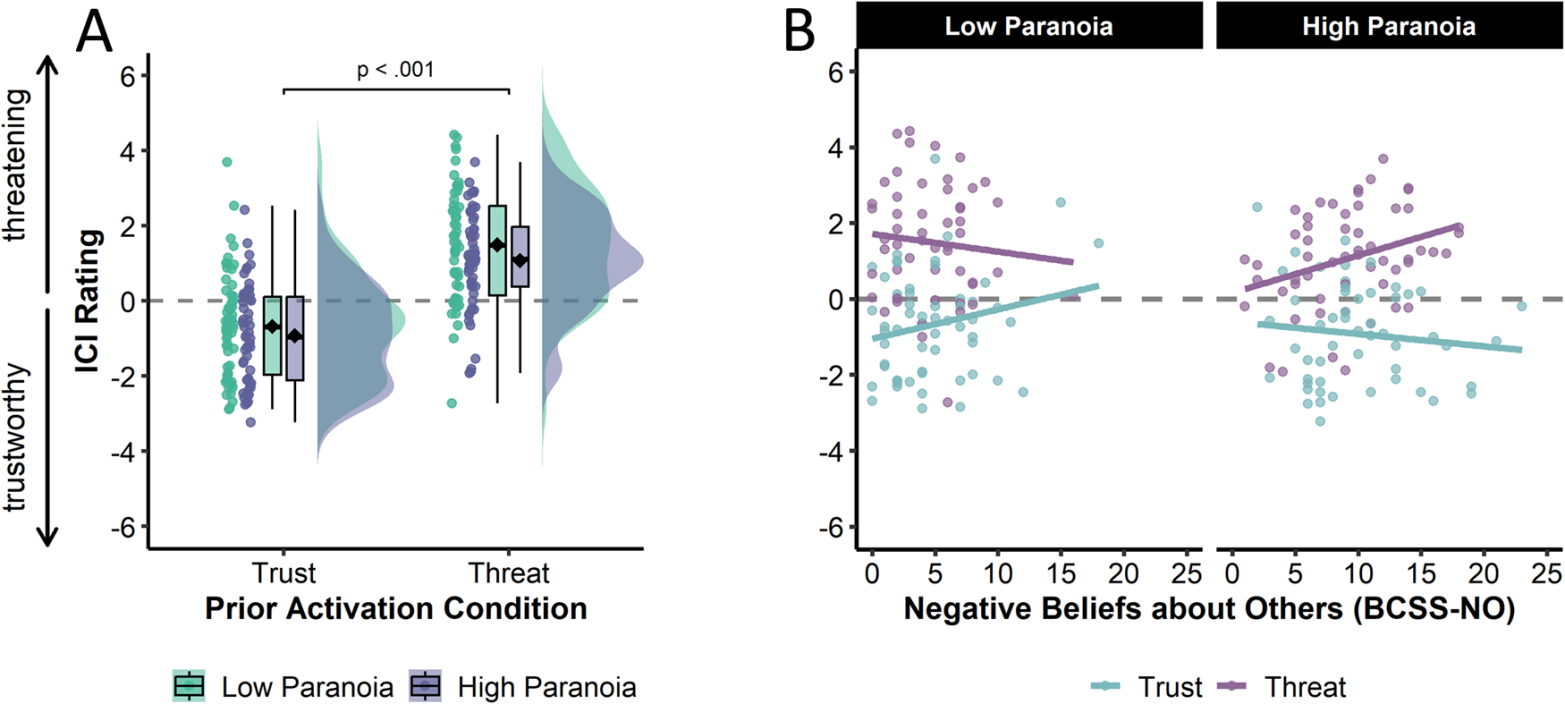
Individual composite image ratings (ICI threat scores). **A** Raincloud plots by condition and paranoia level. Center lines represent medians, diamonds represent mean values, boxes represent the first and third quartiles, and whiskers represent 1.5 × interquartile range. **B** Scatter plots of ICI threat scores as a function of negative beliefs about others. Fitted regression lines are displayed over the data.

The two-way ANOVA revealed a significant main effect of condition on ICI ratings, indicating a higher ICI threat score in the threat condition (*M* = 1.28, *SD* = 1.42) relative to the trust condition (*M* = −0.80, *SD* = 1.36; *F*(1, 216) = 123.05, *p* < 0.001, ${{\rm{\eta }}}_{p}^{2}$ = 0.36, 95%CI [0.26, 0.46], **BF**_**incl**_ = 2.44 × 10^14^; see Fig. 3A). Average ratings of the ICIs generated by participants in the threat condition were significantly above zero (i.e., threatening appearance, *t*(109) = 9.44, *p* < .001, *d* = 0.90, 95%CI [0.68, 1.12]), whereas average ratings of the ICIs generated by participants in the trust condition were significantly below zero (i.e., trustworthy appearance, *t*(109) = −6.12, *p* < .001, *d* = −0.58, 95%CI [−0.78, −0.38]). However, we found no evidence for a main effect of paranoia level (*F*(1, 216) = 3.18, *p* = 0.076, ${{\rm{\eta }}}_{p}^{2}$ = 0.01, 95%CI [0.00, 0.06], **BF**_**incl**_ = 0.56) or an interaction effect between both factors (*F*(1, 216) = 0.23, *p* = 0.632, ${{\rm{\eta }}}_{p}^{2}$ = 0.00, 95%CI [0.00, 0.03], **BF**_**incl**_ = 0.92).

Adding the BCSS-NO and social adversity scores as well as all first- and second-order interactions as covariates to the model did not affect the main and interaction effects. However, the ANCOVA revealed a significant three-way interaction of condition, paranoia, and BCSS-NO (*F*(1,202) = 10.66, *p* = 0.001, ${{\rm{\eta }}}_{p}^{2}$ = 0.05, 95%CI [0.01, 0.12]; see Fig. 3B). Specifically, BCSS-NO and ICI threat scores correlated positively only for HP participants in the threat condition (*r* = 0.32, 95%CI [0.07, 0.54], *t*(54) = 2.51, *p* = 0.015). All results remained the same when controlling for participant age, education, state negative affect, general psychopathology, completion duration, and a metric quantifying the signal in each ICI^64^ (see Supplementary Information S6, S7).

### Exploratory Analyses

We repeated the main analyses including only those HP participants with R-GPTS values ≥ 18 (*n* = 44) to examine whether the expected main and interaction effects would emerge only in participants with at least *severe* paranoia^49^. However, this was not the case (see Supplementary Information S8).

## Discussion

We investigated whether the impact of threat prior beliefs on face perception is stronger in individuals with high versus low paranoia, and whether this effect generalizes to trustworthiness prior beliefs. As expected, we observed a substantial main effect of prior activation condition on ICI appearance, such that ICIs were rated as more threatening in the threat condition and more trustworthy in the trustworthiness condition. However, we neither observed a main effect of paranoia nor an interaction between paranoia level and condition. A more nuanced analysis revealed a significant three-way interaction, indicating that in the threat condition, ICIs generated by participants with high paranoia were rated the more threatening the more strongly they held negative beliefs about others. Social adversity experiences did not affect ICI ratings in either condition.

Our findings conceptually replicate previous evidence documenting a causal effect of behavioral descriptions on ICI appearance^47^. This effect is reflected in the averaged classification images, where the threat condition corresponded to an angry appearance and the trustworthiness condition was associated with a happy appearance, aligning with the established link between emotional valence and social attributions^48^. It is essential to keep in mind that participants were presented with random variations of a group-ambiguous base face and asked to select the faces they believed to best represent a *Group X member*, without explicit assessment of threatening or trustworthy appearances. Consequently, participants could have relied on the face stimuli presented along with the behavioral descriptions during prior activation to inform their mental representations of the groups and, ultimately, stimulus selection. However, our results are consistent with the idea that individuals drew spontaneous trait inferences from the behavioral descriptions (a robust phenomenon; see^65^ for a meta-analysis) and used their mental representations associated with these traits to inform their prior beliefs, guiding subsequent stimulus selection. This aligns with both previous research documenting the effect of several top-down biases (e.g., gender, ethnicity, personality traits) on face perception^46^ and with the fundamental principles of predictive processing, positing that prior beliefs shape the perception of ambiguous sensory inputs^16,46^.

Building on predictive processing accounts of psychosis, which propose an imbalanced integration during perceptual inference as a candidate mechanism underlying delusions^22–24^, we investigated the impact of threat versus trustworthiness prior beliefs on face perception in individuals with high versus low paranoia. As such, we expanded on previous signal detection research utilizing non-social sensory inputs (e.g., rotating dot spheres), acknowledging the predominantly social nature of delusions in general and the specific threat-related social valence in paranoia. Contrary to expectations, ICIs generated by participants with high paranoia were not rated as more threatening or less trustworthy than those generated by participants with low paranoia. Thus, our findings do not support the notion that paranoia relates to overly strong threat prior beliefs that shape the perception of ambiguous facial inputs. A plausible interpretation of this null result could be that paranoid beliefs typically transcend observable behaviors, such as facial expressions, and instead involve the assumption of hidden harmful intentions. Consequently, the threat prior beliefs relevant to paranoia might operate on a higher cognitive level rather than directly impacting low-level perceptual processing^23,24^. These higher-level threat prior beliefs could hinder neutral or even trustworthy facial appearances from being interpreted as evidence against harmful intentions (e.g., ’Others are dangerous, regardless of their facial appearance’ or even ‘I know they have it in for me because they smile, luring me into believing that everything is fine’). In this case, threat prior beliefs might be more evident in the overall evaluation of others rather than in the expectation of threatening facial appearances. Importantly, this alternative explanation remains untested in the present study, offering an intriguing avenue for exploration in future research.

In light of the absence of a paranoia main effect, our findings challenge the notion that delusional beliefs are rooted in a *domain-general* aberrant perceptual inference process. Recent extension to predictive processing accounts of delusion formation suggested that the predominantly social nature might be accommodated through exposure to early social stressors and adversity^66^. Consistent with this idea, ICIs generated by individuals with high paranoia in the threat condition were rated the more threatening the more strongly these individuals held generalized negative beliefs about others. Thus, negative beliefs about others formed throughout life may sensitize individuals with high paranoia to potential threats, intensifying their reliance on threat information and ultimately sculpting their percept into conformity with these threat prior beliefs. While this interpretation is in line with the association between social adversity and psychotic experiences^11,12^ via learned negative beliefs about others^13^, it should be taken with a grain of salt given both the small effect size and the fact that there was no effect of social adversity experiences on ICI threat ratings. Nevertheless, our findings underscore the importance of considering potential influencing factors in investigations of aberrant perceptual inference processes in future research, contributing to a nuanced understanding of the mechanisms underlying delusions.

An alternative explanation relates to the paranoia severity reported by our high paranoia sample, which may not have been elevated enough to observe *overly strong* threat prior beliefs. However, we included only participants who reported at least *moderately severe* paranoia during the last month – a severity that was found optimal to discriminate patients with paranoid delusions from a non-clinical group^49^. Moreover, an exploratory analysis including only participants with *severe* levels of paranoia did not support this interpretation. Therefore, we are confident that our results are not attributable to a lack of paranoia severity in the present sample.

To our knowledge, this study is the first to investigate whether paranoia is linked to strong threat prior beliefs shaping the perception of ambiguous social sensory inputs. We leveraged a well-established social-psychological paradigm to address the challenge of accessing individuals’ prior beliefs while accounting for the social valence inherent in most delusional beliefs. It is noteworthy that participants in this paradigm are typically unaware of the criteria guiding their face selections^46^. The main contribution of this study thus lies in paving the way for investigations into *socially meaningful and potentially incommunicable* prior beliefs, which we believe is crucial when aiming to uncover the mechanisms underlying delusions. Nonetheless, several limitations merit consideration. Firstly, we compared two behavioral description conditions with opposite valences. Therefore, it remains unclear if our results reflect specific traits (threat vs. trustworthiness) or mere valence (negative vs. positive). Importantly, the proposed mechanisms and their interpretations pertain to both scenarios. Secondly, the ICIs represent an approximation of participants’ mental representations, constrained by stimulus materials and performance^46^ as well as the traits rated by the external sample. Future research could employ novel reverse correlation techniques^67^ to test whether our results replicate with photorealistic portraits, potentially incorporating additional personality dimensions of interest. Thirdly, while prior activation may have diminished during RCIC, controlling for individual differences in completion duration revealed no influence of timing on the results. Fourthly, the online setting may have affected performance, although controlling for negative affective states in the beginning of the study did not alter the results. Future studies could repeat the experiment in a more controlled setting such as a laboratory. Lastly, paranoia level was a quasi-experimental factor and the samples significantly differed in age and educational level, potentially limiting the generalizability of the results; however, our results remained robust to controlling for these socio-demographic variables.

In conclusion, our findings suggest that behavioral descriptions inform individuals’ facial prior beliefs, shaping the subsequent perception of others’ faces. Contrary to expectations, our study does not support the idea of a generally stronger impact of threat prior beliefs on face perception in individuals with high paranoia compared to low paranoia. This challenges the assumption that paranoid beliefs operate on a perceptual level. Future research should further investigate the nuanced interplay between threat prior beliefs at different hierarchical levels.

### Supplementary information

Supplemental Material

## Data Availability

The data that support the findings of this study are available at the OSF (https://osf.io/385sy).

## Code availability

The R code that reproduces the present analyses is publicly available at the OSF (https://osf.io/385sy).
