# Supplementary material for: Visualizing threat and trustworthiness prior beliefs in face perception in high versus low paranoia
Source: Schizophrenia (Heidelb). 2024 Mar 20;10(1):40. doi: 10.1038/s41537-024-00459-z (PMC10954723; doi:10.1038/s41537-024-00459-z)
Supplement: Supplementary file 1 — Supplemental Material [file 41537_2024_459_MOESM1_ESM.pdf]

Supplementary Information

Visualizing Threat and Trustworthiness Prior Beliefs in Face Perception in High versus Low Paranoia

Antonia Bott[1]*, Hanna C. Steer[1], Julian L. Faße[1], and Tania M. Lincoln[1]

[1]Clinical Psychology and Psychotherapy, Faculty of Psychology and Human Movement Science,

Universität Hamburg, Germany

*Corresponding author: E-mail: antonia.bott@uni-hamburg.de

Table of Contents

S1: Stimuli Used for Prior Activation and Reverse Correlation Image Classification 2

S2: RCIC Strategy use 7

S3: Deviations from Preregistered Procedure 8

S4: Bayesian Analysis Results 9

S5: Sample Differences in Self-report Measures 13

S6: ANCOVA Results Including Demographic Covariates 14

S7: ANCOVA Results Including BCSS-NO and SAE as Covariates 15

S8: ANOVA Results of Individuals with Severe Levels of Paranoia 17

S9: Face Rating.. 18

S10: Correlations among Raters' Paranoia and ICI Ratings 20

References 21

S1: Stimuli Used for Prior Activation and Reverse Correlation Image Classification

Prior activation

Within the prior activation phase, participants viewed 30 face stimuli paired with behavioral descriptions (i.e., 15 per fictitious group). We created the group-specific base faces by randomly selecting 10 male faces per group (20 faces in total) from the Chicago Face Database (CFD[1]). These selections included WM-001-014-N, WM-003-002-N, WM-004-010-N, WM-011-002-N, WM-018-002-N, WM-023-001-N, WM-031-003-N, WM-206-045-N, WM-219-008-N, and WM-232-070-N (first base face), as well as WM-006-002-N, WM-016-001-N, WM-020-001-N, WM-033-025-N, WM-035-032-N, WM-204-031-N, WM-205-007-N, WM-210-057-N, WM-229-129-N, and WM-230-131-N (second base face). We used the validation data[1] published along with the CFD to ensure that these face selections did not differ significantly in perceived age, anger, happiness, threat, trustworthiness, dominance, attractiveness, and masculinity. As can be seen in Table S1-1, this was not the case. Next, we used *Adobe Photoshop* to morph the 10 faces per group, converted the resulting morphs to grayscale, cropped them to 512 × 512 pixels and smoothed them with a Gaussian blur (kernel size: 0.6 pixels; see Fig. s S1-1 and S1-2, top left image). Finally, we superimposed each group-specific base face with 15 unique patterns of sinusoidal visual noise (random seeds set to 13 and 26, respectively) by using the rcicr package[2] for R (see Fig. S1-1 and S1-2 for a comprehensive collection of face stimuli used during prior activation).

The resulting 15 face stimuli per group were presented along with unique behavioral descriptions, ten of which either implied the trait *threatening* (e.g., “This member of Group X spies on you”) or *trustworthy* (e.g., “This member of Group Y keeps a secret you told him”) and five of which depicted *neutral* behaviors (e.g., “This member of Group X happens to wait with you at the train station”). Generation of the *threat-implying* behavioral descriptions was guided by items typically used in scales assessing paranoid beliefs (e.g., Paranoia Checklist[3]), while the *trustworthiness-implying* behavior descriptions were phrased complementarily in order to offset the threatening behaviors

(similar to[4]). The neutral items were used as distractors. The assignment of base faces to Group X and Group Y, the order of block-wise group presentation (i.e., Group X vs. Group Y first), as well as the order of face-behavior pairs within blocks were randomized. Face-behavior pairs were random but fixed across subjects. Stimulus pairs were implemented via *Qualtrics* (www.qualtrics.com), and task completion was self-paced (*Mdn* = 8.53 min, *SD* = 8.08) with a minimum stimulus presentation duration of 5 sec.

Table S1-1

Comparisons of the face stimuli used to create the group-specific base faces for prior activation.

Variable	n_1	n_2	statistic[a]	*df*	*p*
Age	10	10	-0.343	17.139	0.736
Angry	10	10	-0.699	17.387	0.494
Attractive	10	10	-0.43	15.931	0.673
Dominant	10	10	-1.184	17.653	0.252
Happy	10	10	0.578	17.948	0.571
Masculine	10	10	-0.713	17.861	0.485
Threatening	10	10	-0.913	17.718	0.374
Trustworthy	10	10	1.144	17.627	0.268

Note. [a] Welch two-sample t-test

Figure S1-1

First base face (top left image) superimposed with 15 random sinusoidal noise patterns used during prior activation (all other images).

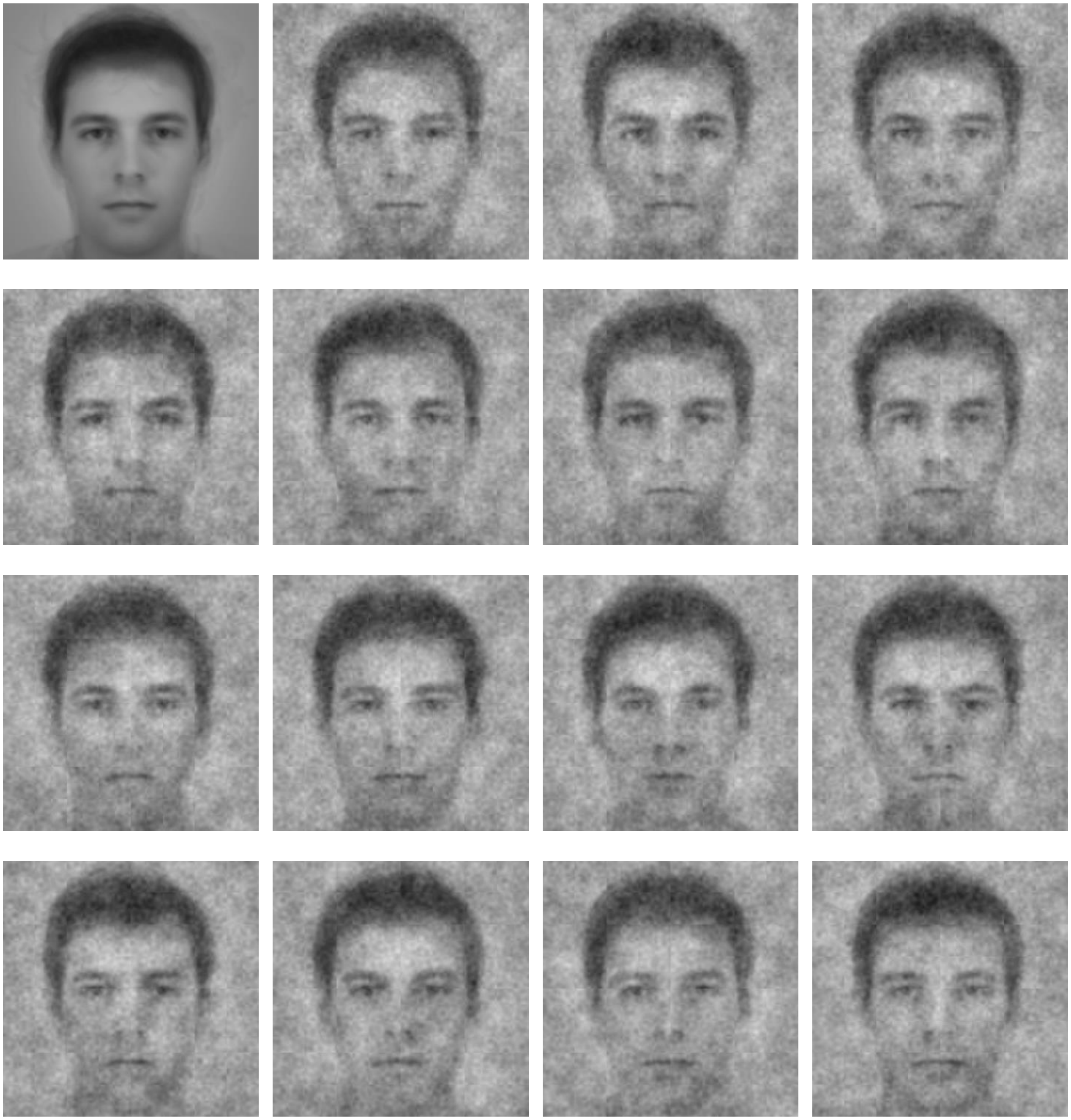

Figure S1-2

Second base face (top left image) superimposed with 15 random sinusoidal noise patterns used during prior activation (all other images).

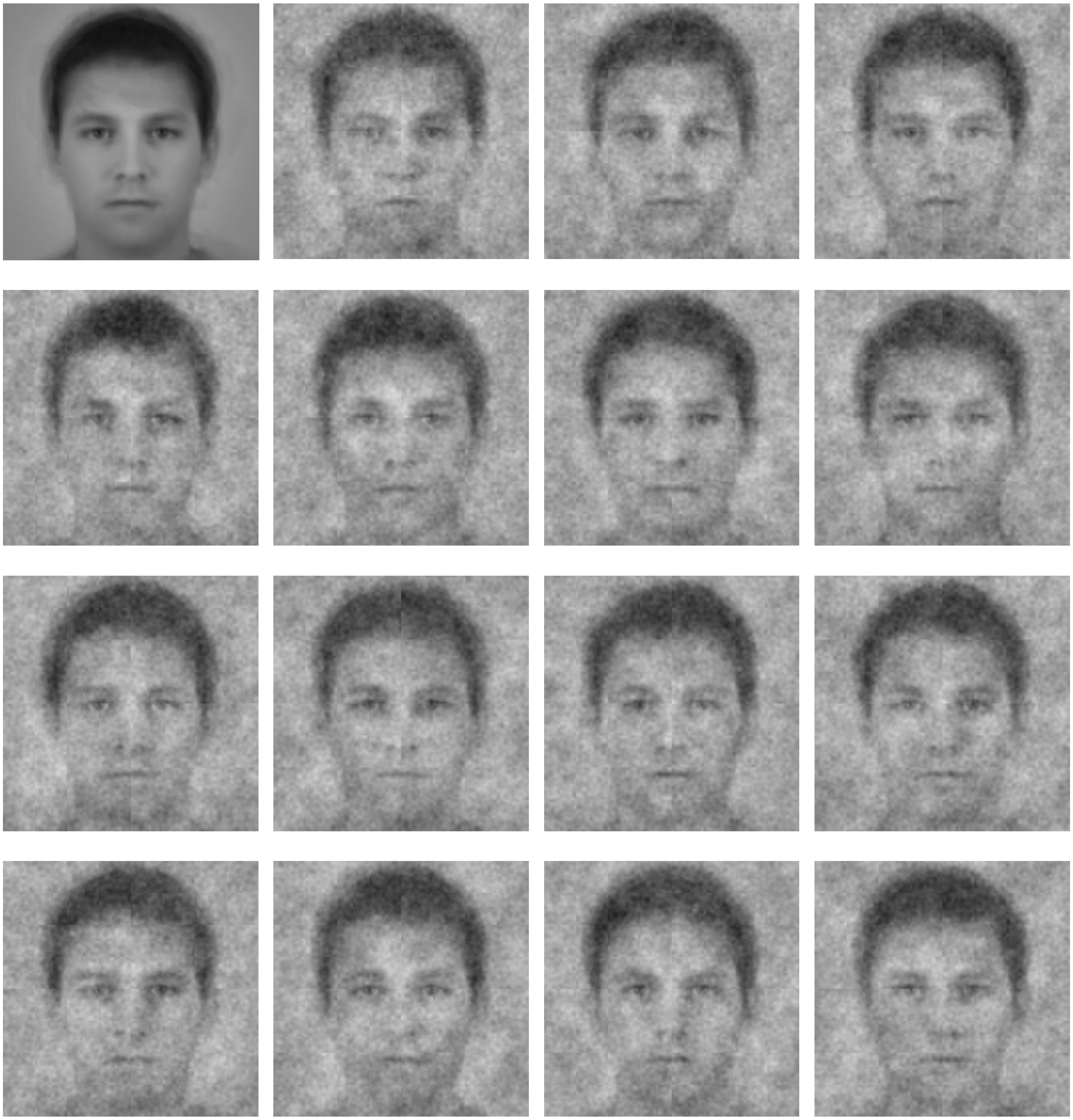

Reverse Correlation Image Classification Paradigm Stimulus Generation

We created one group-ambiguous base face by using Adobe Photoshop to morph the group-specific base faces used during prior activation. Next, we generated 400 stimulus pairs by superimposing this base face with both unique random sinusoidal noise patterns and their mathematical inverses (a white pixel in the original noise pattern is black in its inversion and vice versa, see Figure S1-3) using the `generateStimuli2IFC()` from the rcicr package (random seed set to 10)[2]. Stimulus pairs were identical for all participants, but presentation order as well as position on the screen (left vs. right) were randomized. The experiment was implemented by adapting the Processing (www.processing.org) code provided by Anton Gollwitzer (www.github.com/AntonGollwitzer/ReverseCorrelationRunningOnline) to the present research focus. In each trial, one pair of stimuli was presented side-by-side against a black background (512 × 512 pixels), with the categorization item “Wer gehört zu Gruppe X?” (“Who belongs to Group X?”) displayed above and a progress percentage displayed below the stimuli (white font color). Task completion was self-paced (*Mdn* = 16.59 min, *SD* = 13.16).

Figure S1-3

Sample RCIC stimulus pair, superimosed with random noise and the inverted noise pattern

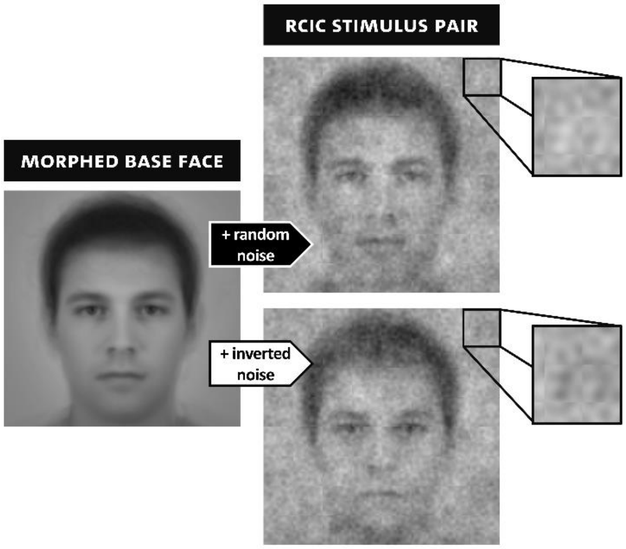

S2: RCIC Strategy use

We coded participants' open responses regarding their strategy use during RCIC task completion in categories. In total, 136 participants (62%) reported having used a specific strategy. Most frequent responses included focusing on facial features (mainly the eye and mouth region; n = 59, 27%), identifying specific facial features signaling positive or negative valence (e.g., 'aggressive gaze'; n = 27, 12%), and looking for positive or negative valence in the faces without naming specific features (e.g., 'looking trustworthy'; n = 36, 16%). 9 participants (4%) reported having focused on other physical attributes (e.g., masculinity) or similarity to known persons. Responses of 5 participants (2%) did not apply to any category and were coded 'other'; 84 participants (38%) did not report a strategy.

S3: Deviations from Preregistered Procedure

- *Explicit evaluation of Group X and Group Y*: Given the empirical non-normality of the explicit group evaluation difference score, we deviated from our pre-registered analysis plan in that we used a Wilcoxon rank-sum test instead of a one-way ANOVA to test for differences between conditions. Moreover, we added a Wilcoxon rank-sum test to test for differences between HP and LP samples, which we had not preregistered, to probe whether the prior manipulation was equally effective across paranoia levels.
- *Exclusion due to RCIC responses:* In addition to the pre-registered exclusion criterion (i.e., click one stimulus in 95% of the trials; did not apply to any participant), we applied three additional exclusion criteria we had not preregistered and excluded n = 4 participants based on these criteria. (1) Due to a programming error of the RCIC paradigm we became aware of after data collection was complete, participants could also proceed to the next trial by clicking on the margins (i.e., not on one of two stimuli, but on the black background left, right, up, down, and in between the stimuli). We excluded two participants (both LP sample, threat condition) who clicked the margin *in between* the stimuli in a significant number (i.e., 25% and 42%) of the trials, given that we could not infer which stimulus they had intended to select for these trials. (2) One participant (LP sample, trust condition) was excluded because of a RCIC completion duration of >19 h given that we did not expect this participant to maintain the associations learned during the prior activation phase across this time. (3) Due to a programming error of the ICI rating task, one participant's ICI was not rated, so that we had to exclude them from further analyses (HP sample, trust condition). All exclusion criteria were applied before any analyses related to the main outcome variables have been conducted.

S4: Bayesian Analysis Results

We complemented our manipulation check and main analyses with Bayes Factors (BF) obtained from Bayesian analysis counterparts performed with JASP [5]. These analyses were not preregistered. BF hypothesis testing differs from hypothesis testing using the *p*-value in that it directly and continuously compares the predictive adequacy of two competing statistical models (i.e., the null hypothesis and the alternative hypothesis), thus quantifying the relative evidence for and change brought about each of these models after seeing the data (for an overview, see[6]). Specifically, BF_{10} (and its inverse $BF_{01} = 1/BF_{10}$) quantifies the intensity of evidence for the alternative hypothesis H_1 versus the null hypothesis H_0 (and vice versa). According to a rule of thumb guideline [7], a BF_{10} between 1 and 3 can be interpreted as weak evidence, a BF_{10} between 3 and 10 as moderate, and a BF_{10} greater than 10 as strong evidence for the alternative as compared to the null hypothesis.

Manipulation check

As expected, the results of a two-sided Bayesian Mann-Whitney U test (also referred to as Wilcoxon rank sum test) across conditions indicated that the data were $3.48\times10^{+8}$ times more likely under H_1 than H_0 (see Table S4-1), yielding strong evidence for the hypothesis that participants in the threat condition rated Group X more negatively than Group Y (whereas the opposite was true for the trust condition, see Table S4-2). By contrast, a two-sided Mann-Whitney U test across paranoia levels suggested the data were approximately 6 times more likely under H_0 than under H_1, yielding moderate evidence for the hypothesis that explicit group evaluations did not differ across paranoia levels (see Figure S4-1).

Table S4-1

Results of Bayesian Mann-Whitney U Tests comparing explicit group evaluation across paranoia levels and conditions

Factor	BF_{10}	*W*	Rhat
Paranoia level	0.15	5951.00	1.00
Condition	$3.48 \times 10^{+8}$	411.00	1.01

Note. Result based on data augmentation algorithm with 5 chains of 1000 iterations.

Table S4-2

Descriptive statistics of explicit group evaluations

						95% Credible Interval	
		N	*M*	*SD*	*SE*	Lower	Upper
Paranoia level	HP	109	-0.14	4.48	0.43	-0.99	0.71
	LP	111	-0.04	4.13	0.39	-0.81	0.74
Condition	Threat	110	-3.77	2.03	0.19	-4.16	-3.39
	Trust	110	3.60	2.34	0.22	3.16	4.04

Note. Positive explicit group evaluations indicate that Group X was rated more positively than Group Y, whereas negative values indicate the opposite. LP = low paranoia, HP = high paranoia, SD = standard deviation, SE = standard error.

Figure S4-1

Inferential plots of Bayesian Mann-Whitney U Tests comparing explicit group evaluation between conditions (a) and paranoia levels (b)

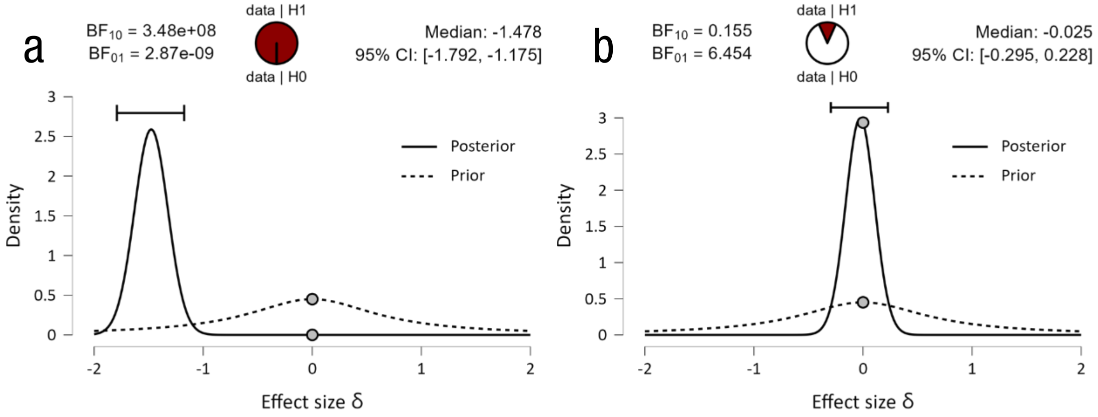

Note. Reference categories were trust (condition) and low paranoia (paranoia level).

Main analyses

We submitted the ICI threat scores to a Bayesian two-way ANOVA with Condition and Paranoia level as between-subjects factors. As can be seen in Table S4-3, the data were most likely under a model considering only Condition as predictor. More specifically, they were approximately 1/0.36 = 2.78 times more likely than under a model also including participants' paranoia level (additively) and 1/0.08 = 12.5 times more likely than under a model additionally including the interaction between both factors. The model-averaged analysis of effects (see Table S4-4) suggested that there was strong evidence for including Condition as a predictor ($BF_{incl} = 2.73 \times 10^{+14}$), whereas the evidence in the data for including Paranoia level as well as the interaction between both factors was anecdotal. Table B-5 shows a summary of the marginal model-average posterior distributions. In sum, the results of our Bayesian analysis counterparts converged with the results of the frequentist approach.

Table S4-3

Results of Bayesian model comparison with the best model as reference

Models	P(M)	P(M|data)	BF_M	BF_{10}	error %
Condition	0.20	0.56	5.05	1.00	
Condition + Paranoia level	0.20	0.36	2.25	0.64	1.05
Condition + Paranoia level + Condition × Paranoia level	0.20	0.08	0.36	0.15	1.44
Null model	0.20	1.32×10^{-20}	5.28×10^{-20}	2.37×10^{-20}	3.59×10^{-26}
Paranoia level	0.20	3.76×10^{-21}	1.50×10^{-20}	6.73×10^{-21}	1.17×10^{-4}

Note. Models = predictors included in each model; P(M) = prior model probability; P(M|data) = posterior model probability; BF_M = posterior model odds; BF_{10} = Bayes factors of all models compared to the best model; error % = estimate of the numerical error in the computation of the Bayes factor, with errors below 20% being acceptable in many cases.

Table S4-4

Model-averaged analysis of effects

Effects	P(incl)	P(excl)	P(incl|data)	P(excl|data)	BF_{incl}
Condition	0.60	0.40	1.00	2.44×10^{-15}	$2.73\times10^{+14}$
Paranoia level	0.60	0.40	0.44	0.56	0.53
Condition × Paranoia level	0.20	0.80	0.08	0.92	0.36

Note. P(incl) = prior inclusion probability; P(excl) = Prior exclusion probability; P(incl|data) = posterior includion probability; P(excl|data) = posterior exclusion probability; BF_{incl} = inclusion Bayes factor which can be interpreted as evidence in the data for including a predictor.

Table S4-5

Summary of the marginal model-averaged posterior distributions

				95% Credible Interval	
Variable	Level	*M*	*SD*	Lower	Upper
Intercept		0.24	0.09	0.05	0.43
Condition	Threat	1.03	0.10	0.83	1.21
	Trust	-1.03	0.10	-1.22	-0.83
Paranoia level	HP	-0.16	0.09	-0.34	0.02
	LP	0.16	0.09	-0.03	0.34
Condition × Paranoia level	Threat × HP	-0.04	0.09	-0.22	0.14
	Threat × LP	0.04	0.09	-0.14	0.22
	Trust × HP	0.04	0.09	-0.14	0.22
	Trust × LP	-0.04	0.09	-0.22	0.14

Note. LP = low paranoia, HP = high paranoia.

S5: Sample Differences in Self-report Measures

Figure S5-1

Boxplots of self-report measures by paranoia level

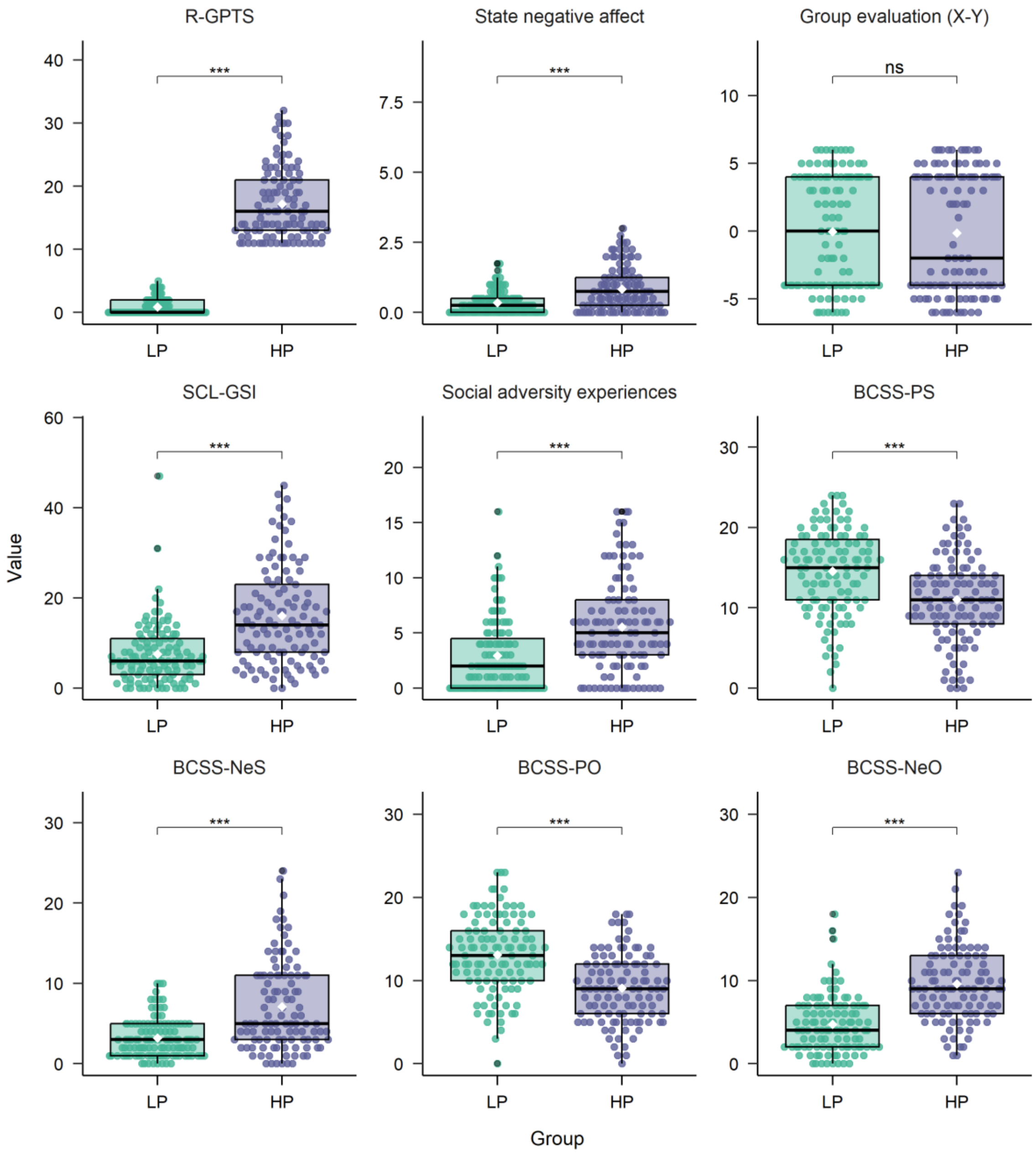

Note. BCSS=Brief Core Schema Scale, PS=Positive Self, NeS=Negative Self, PO=Positive Others, NeO=Negative Others, LP=Low paranoia, HP=high paranoia, R-GPTS=Revised Green et al., Paranoid Thoughts Scale, persecution, SCL-GSI=Symptom Checklist, General severity index.

S6: ANCOVA Results Including Demographic Covariates

Table S6-1

Results of ANCOVA on ICI threat score including demographic covariates, completion duration, and individual informational values of the ICIs.

Predictor	df	SS	MS	F	p
Condition	1.00	239.68	239.68	122.06	**< .001**
Paranoia level	1.00	5.63	5.63	2.87	.092
Age	1.00	0.01	0.01	0.01	.939
Education	2.00	0.20	0.10	0.05	.950
State-NA	1.00	0.29	0.29	0.15	.701
SCL-14	1.00	1.42	1.42	0.73	.395
Duration (min)	1.00	2.90	2.90	1.48	.226
InfoVal	1.00	0.56	0.56	0.29	.593
Condition × Paranoia level	1.00	0.91	0.91	0.47	.496
Residual	208.00	408.53	1.96		

Note. State-NA = State negative affect, SCL-14 = Symptom Checklist, general severity index, InfoVal = Informational Value.

S7: ANCOVA Results Including BCSS-NO and SAE as Covariates

Table S7-1

Results of ANCOVA on ICI threat score including negative beliefs about others, social adversity experiences, and demographic control variables as covariates

Predictor	df	SS	MS	F	p
Condition	1.00	239.68	239.68	129.29	**< .001**
Paranoia level	1.00	5.63	5.63	3.04	.083
BCSS-NO	1.00	3.39	3.39	1.83	.178
SAE	1.00	1.38	1.38	0.74	.390
Age	1.00	0.01	0.01	0.00	.953
Education	2.00	0.08	0.04	0.02	.980
State-NA	1.00	0.07	0.07	0.04	.842
SCL-14	1.00	6.88	6.88	3.71	.056
Duration (min)	1.00	2.66	2.66	1.43	.233
InfoVal	1.00	0.24	0.24	0.13	.719
Condition × Paranoia level	1.00	1.05	1.05	0.56	.453
Paranoia level × BCSS-NO	1.00	0.01	0.01	0.01	.938
Condition × BCSS-NO	1.00	1.23	1.23	0.66	.417
Paranoia level × SAE	1.00	4.14	4.14	2.23	.137
Condition × SAE	1.00	0.36	0.36	0.19	.661
Condition × Paranoia level × BCSS-NO	1.00	19.40	19.40	10.46	**.001**
Condition × Paranoia level × SAE	1.00	3.18	3.18	1.72	.192
Residual	200.00	370.77	1.85		

Note. BCSS-NO = Brief Core Schema scale, Negative others, SAE = Social adversity experiences, SCL-14 = Symptom Checklist (14-item variant), State-NA = negative affective states, InfoVal = Informational Value.

Figure S7-1

Association between ICI threat scores (threat – trustworthiness rating) and social adversity per Paranoia level and Condition

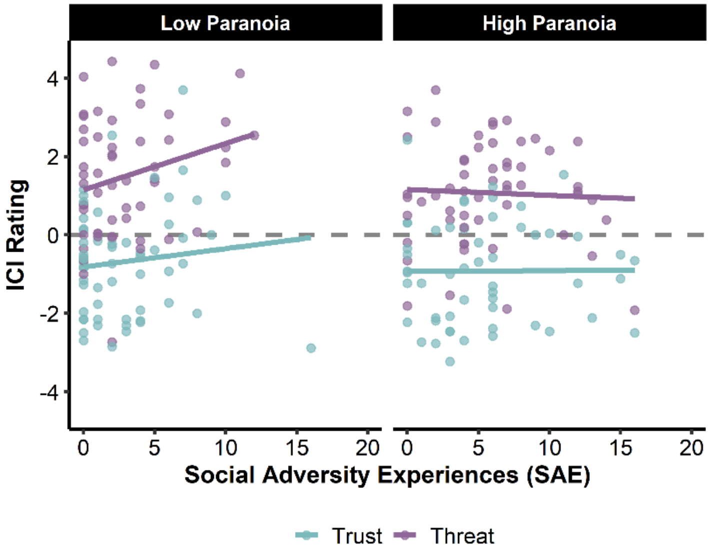

S8: ANOVA Results of Individuals with Severe Levels of Paranoia

Table S8-1

Results of two-way ANCOVA on ICI threat score including only individuals with severe levels of paranoia, controlled for demographic covariates

Predictor	df	SS	MS	F	p
Condition	1.00	176.41	176.41	88.65	**< .001**
Paranoia level	1.00	4.09	4.09	2.06	.154
Age	1.00	0.14	0.14	0.07	.795
Education	2.00	1.57	0.78	0.39	.675
State-NA	1.00	0.16	0.16	0.08	.776
SCL-14	1.00	1.17	1.17	0.59	.444
Condition × Paranoia level	1.00	0.33	0.33	0.16	.686
Residual	145.00	288.54	1.99		

Note. N = 155, high paranoia sample with R-GPTS score higher than or equal to 18. State-NA = State negative affect, SCL-14 = Symptom Checklist, general severity index

S9: Face Rating

Participants of the first stage of the study (i.e., ICI generators) rated 10 human portraits with neutral emotional expression on trust, dominance, threat, and attractiveness (the latter being a distractor item) using 7-point scales (ranging from *1: not at all* to *7: very*) before completing the self-reports. The faces were drawn randomly from the Chicago Face Database (Ma et al., 2015; www.chicagofaces.org) and included five male faces (i.e., WM-015-002-N, WM-205-007-N, WM-214-026-N, WM-220-068-N, WM-232-070-N) and five female faces (i.e., WF-011-002-N, WF-022-017-N, WF-033-002-N, WF-217-085-N, WF-237-067-N).

We compared face ratings per trait across paranoia levels using Welch two sample *t* tests. Replicating previous findings[8], participants with high levels of paranoia rated male faces as significantly more threatening (and descriptively as less trustworthy and more dominant) than participants with low levels of paranoia (see Table S9-1). Interestingly, this was not true for female faces. However, the sample difference in threat ratings were not significant after correcting for multiple testing (α_{adj} = .005).

Table S9-1

Paranoia level differences as well as means and standard deviations of the face ratings.

Rating	Low Paranoia		High Paranoia		
	M	(*SD*)	*M*	(*SD*)	Welch Two Sample t-test[1]
Female faces (k = 5)					
trustworthiness	4.02	0.74	4.11	0.85	$t(212.95) = 0.89, p = .372$
threat	2.71	0.86	2.86	0.88	$t(217.61) = 1.29, p = .200$
dominance	3.82	0.81	3.85	0.85	$t(217.04) = 0.20, p = .841$
attractiveness	3.81	0.96	3.95	0.90	$t(217.63) = 1.13, p = .260$
Male faces (k = 5)					
trustworthiness	3.64	0.73	3.54	0.74	$t(217.82) = -1.09, p = .279$
threat	3.52	0.81	3.76	0.85	$t(217.28) = 2.22, p = .028$
dominance	3.79	0.79	3.97	0.81	$t(217.67) = 1.62, p = .108$
attractiveness	3.00	0.93	3.07	0.94	$t(217.82) = 0.57, p = .570$

Note. [1] = uncorrected p-values.

S10: Correlations among Raters' Paranoia and ICI Ratings

Given the association between paranoia and a bias in face ratings (i.e., individuals with high paranoia tend to rate faces as angrier and more dominant as well as less trustworthy), we tested whether raters' self-reported paranoia correlated with their mean ratings of the individual classification images (ICIs). However, raters' R-GPTS sum scores were not correlated with ICI trustworthiness ratings (r = .09, 95% CI [-.14, .31], $t(74)$ = 0.76, p = .448) or ICI threat ratings (r = .06, 95% CI [-.17, .28], $t(74)$ = 0.53, p = .594). Thus, we averaged ICI trustworthiness and threat ratings across raters to calculate the outcome variable.

References

1. Ma, D. S., Correll, J. & Wittenbrink, B. The Chicago face database: A free stimulus set of faces and norming data. *Behav. Res. Methods* **47**, 1122–1135 (2015).
2. Dotsch, R. rcicr: Reverse correlation image classification toolbox. (2017).
3. Freeman, D. *et al.* Psychological investigation of the structure Psychological investigation of the structure of paranoia in a non-clinical population. *Br. J. Psychiatry* **186**, 427–435 (2005).
4. Dotsch, R., Wigboldus, D. H. J. & Van Knippenberg, A. Behavioral information biases the expected facial appearance of members of novel groups. *Eur. J. Soc. Psychol.* **43**, 116–125 (2013).
5. JASP Team. JASP. (2022).
6. Wagenmakers, E.-J. *et al.* Bayesian inference for psychology. Part I: Theoretical advantages and practical ramifications. *Psychon. Bull. Rev.* **25**, 35–57 (2018).
7. Kass, R. E. & Raftery, A. E. Bayes Factors. *J. Am. Stat. Assoc.* **90**, 773–795 (1995).
8. Trémeau, F. *et al.* What can the study of first impressions tell us about attitudinal ambivalence and paranoia in schizophrenia? *Psychiatry Res.* **238**, 86–92 (2016).
